# Synthetic Biology Strategies and Tools to Modulate Photosynthesis in Microbes

**DOI:** 10.3390/ijms26073116

**Published:** 2025-03-28

**Authors:** Shujin Fu, Kaiyu Ma, Xinyu Song, Tao Sun, Lei Chen, Weiwen Zhang

**Affiliations:** 1 Frontier Science Center for Synthetic Biology and Key Laboratory of Systems Bioengineering, Ministry of Education of China, Tianjin 300072, China; fushujin@tju.edu.cn (S.F.); kaiyu_ma001@tju.edu.cn (K.M.); tsun@tju.edu.cn (T.S.); lchen@tju.edu.cn (L.C.); 2 Laboratory of Synthetic Microbiology, School of Chemical Engineering & Technology, Tianjin University, Tianjin 300072, China; 3 Tianjin University Center for Biosafety Research and Strategy, Tianjin 300072, China; 4 State Key Laboratory of Synthetic Biology, Tianjin University, Tianjin, 300072, China

**Keywords:** cyanobacteria, microalgae, synthetic biology, molecular biology, photosynthesis efficiency

## Abstract

The utilization of photosynthetic microbes, such as cyanobacteria and microalgae, offers sustainable solutions to addressing global resource shortages and pollution. While these microorganisms have demonstrated significant potential in biomanufacturing, their industrial application is limited by suboptimal photosynthetic efficiency. Synthetic biology integrates molecular biology, systems biology, and engineering principles to provide a powerful tool for elucidating photosynthetic mechanisms and rationally optimizing photosynthetic platforms. This review summarizes recent advancements in regulating photosynthesis in cyanobacteria and microalgae via synthetic biology, focusing on strategies to enhance light energy absorption, optimize electron transport chains, and improve carbon assimilation. Furthermore, we discuss key challenges in translating these genetic modifications to large-scale bioproduction, highlighting specific bottlenecks in strain stability, metabolic burden, and process scalability. Finally, we propose potential solutions, such as AI-assisted metabolic engineering, synthetic microbial consortia, and next-generation photobioreactor designs, to overcome these limitations. Overall, while synthetic biology holds great promise for enhancing photosynthetic efficiency in cyanobacteria and microalgae, further research is needed to refine genetic strategies and develop scalable production systems.

## 1. Introduction

In the face of increasing global resource shortages and environmental pollution, the search for sustainable energy and production methods has become a critical priority [1,2]. Photosynthetic microorganism-based green biomanufacturing offers a promising pathway toward sustainable development [3]. Prokaryotic cyanobacteria and eukaryotic microalgae, two major platforms of photosynthetic microbial cell factory, each have distinct characteristics and potential [4,5]. Eukaryotic microalgae have attracted substantial attention on account of their capacity to flourish in outdoor settings and their high productivity in generating proteins and lipids [6]. In contrast, cyanobacteria, with their well-characterized genetic background and high genetic manipulability, have emerged as more designable and scalable chassis cells for photosynthesis processes [7,8]. By introducing heterologous metabolic pathways and modifying their metabolic networks, cyanobacteria have successfully produced various high-value products, including sucrose [9], inositol [10], 3-HB, 3-HV, 3-HP [11], and astaxanthin [12]. Microalgae also play a significant role in green biomanufacturing, particularly in the production of triglycerides (TAG) [13] and fatty acid esters (biodiesel) [14]. Additionally, they can generate valuable compounds, such as β-carotene [15], astaxanthin [16], lutein [17], and other carotenoids [18]. However, despite these advancements, the productivity of photosynthetic microorganisms remains lower than that of terrestrial plants, which are commonly utilized in biomass-based production systems [19,20]. While algae, cyanobacteria, and plants can all be processed through different types of biorefinery technologies, the inherent limitations of photosynthetic microorganisms, such as lower biomass accumulation and metabolic efficiency, present challenges for their large-scale industrial application. Notably, consortia between microalgae and N-fixing cyanobacteria have demonstrated promising biotechnological potential by enhancing nutrient availability, promoting mutualistic interactions, and potentially, improving overall biomass yield [21].

Photosynthetic microorganisms can capture nearly 50% of incident light energy; however, less than 10% of that energy is capable of being converted into chemical energy presented as biomass. The maximum theoretical solar-to-biomass energy conversion efficiency for oxygenic photosynthesis is estimated to be around 9–10% under ideal conditions [22]. However, in practice, the efficiency is significantly lower, with cyanobacteria achieving approximately 1–2% and microalgae reaching 4–8% [22,23]. This discrepancy is primarily due to various limiting factors, such as inefficient light absorption, suboptimal carbon fixation pathways, and energy losses in metabolic processes. Bridging this gap between theoretical and actual efficiency is crucial for improving the industrial viability of photosynthetic microorganisms in bio-based production systems. Photosynthesis takes place in the following two phases: the light reactions and the dark reactions [24]. During the light reactions, through their light-harvesting antenna system, photosynthetic microorganisms trap solar energy and then convey it to the reaction centers of the photosystem complexes, converting it into electrical energy. Electrons are transferred through various protein complexes and electron carriers within photosynthetic membrane, storing energy as ATP and NAD(P)H. In the dark reactions, ATP and NAD(P)H are used to fix CO_2_ and produce organic compounds. The systematic optimization of three key processes—light energy absorption and utilization, electron transport chains, and carbon assimilation—is widely recognized as an effective strategy for improving photosynthetic efficiency [25].

**Figure 1:**
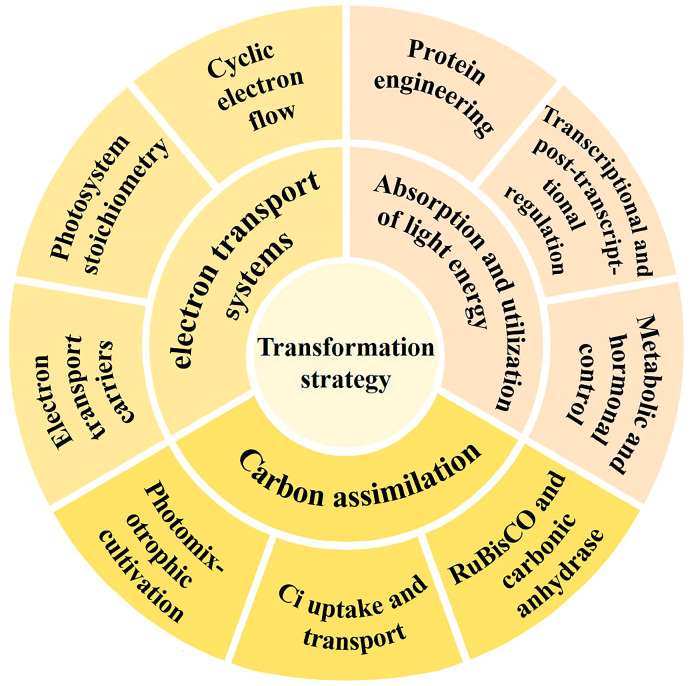
Regulatory targets in photosynthesis. The goals of the transformation strategy are divided into three parts: absorption and utilization of light energy, electron transport systems, and carbon assimilation.

Synthetic biology represents an interdisciplinary domain that synergistically amalgamates biology, genomics, engineering, and informatics [26]. It approaches biological systems as engineered systems using a “bottom-up” methodology, following an iterative “design–build–test–learn” research strategy. This process moves from “units” to “devices” to “systems”, with the goal of redesigning natural organisms or creating new synthetic ones. The ultimate aim is to uncover the principles of life and transform biological systems for engineering applications [27,28,29]. Through synthetic biology, a deeper understanding of photosynthesis in photosynthetic microorganisms can be achieved, enabling the more effective regulation of photosynthetic efficiency. Despite this, several significant challenges remain for applying synthetic biology to regulate photosynthetic microbial cell factories; for example, (i) it is difficult to improve photosynthetic efficiency by modifying just one or a few genes; (ii) many unknown functional proteins impact the regulatory effect; (iii) synchronizing photosynthetic efficiency with cell growth optimization remains challenging; and (iv) the effects of genetic engineering are often difficult to translate into large-scale production processes. In this context, we provide an overview of the work and accomplishments from the past few years in regulating photosynthesis in microalgae and cyanobacterium through synthetic biology strategies (Figure 1), along with the major challenges and future perspectives. The efforts provide a foundation for further deciphering the photosynthesis mechanisms and improving photosynthetic efficiency in these cell systems (Table 1).

## 2. Modulation of Absorption and Utilization of Light Energy

**Figure 2:**
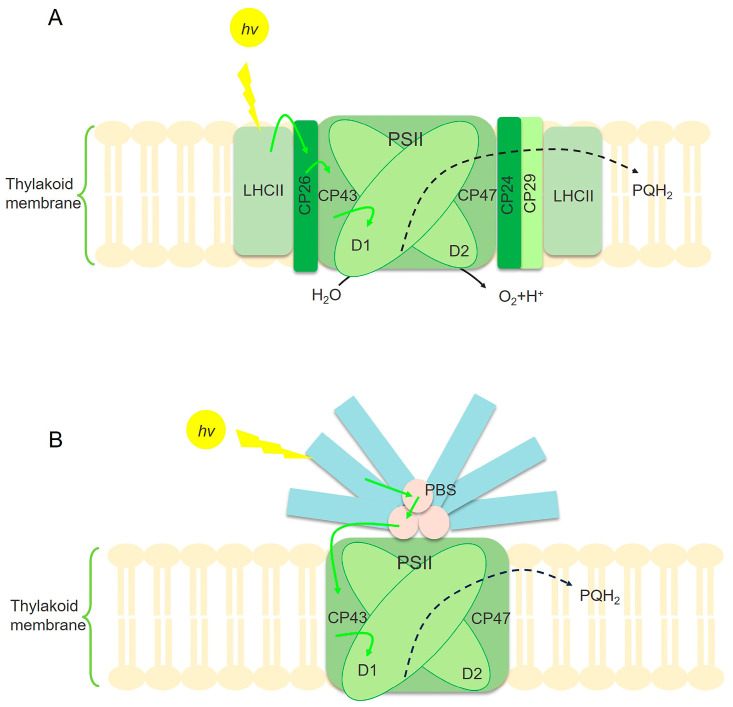
Illustration of the mechanism of light energy absorption and utilization in microalgae (**A**) and cyanobacteria (**B**). PSII is the main site for cyanobacteria and photosynthetic microalgae to absorb light energy. The center of the PSII core is formed by D1 and D2 proteins. CP43 and CP47 are protein complexes that bind to several Chl-a molecules, serving as the inner antenna of PSII. Photosynthetic microalgae utilize the main membrane-integral LHC and some secondary chlorophyll protein complexes to capture sunlight, such as CP24, CP26, and CP29 (**A**). However, the main light-harvesting complexes of PSII in cyanobacteria are PBSs (**B**) [56]. The yellow arrow represents light energy absorption. The green arrow represents the transfer of excitation energy. The black dashed arrow represents electronic transmission.

The initial phase of photosynthesis, designated as the primary reaction, encompasses the sequence of processes commencing from the excitation of chlorophyll molecules upon light absorption and culminating in the initiation of the first photochemical reaction [55]. This stage includes the absorption, transfer, and conversion of light energy by pigment molecules. Regulating the processes—light energy absorption, transfer, and conversion—has become a key strategy for improving the efficiency of light energy utilization (Figure 2). Photosynthetic microalgae and cyanobacteria share comparable internal antenna systems, which consist of protein complexes binding chlorophyll a (Chl-a) for light capture. However, their external light-harvesting pigment–protein complexes (LHCs) differ. In photosynthetic microalgae, the principal external light-harvesting complexes are the membrane-integral LHCII and several minor chlorophyll–protein complexes, which are responsible for capturing sunlight (Figure 2A). In cyanobacteria, the primary external light-harvesting complex is the phycobilisome (PBS). This complex is affixed to the cytoplasmic surface of the PSII reaction centers and assumes a pivotal role in light absorption (Figure 2B) [56]. Optimizing light absorption and utilization involves the following three major regulatory mechanisms: protein engineering, transcriptional control, and metabolic modulation. Below, we discuss recent advances in these regulatory strategies, categorized by their common mechanisms.

### 2.1. Protein Engineering for Light Absorption Optimization

Protein engineering offers a direct approach to modifying light-harvesting complexes for improved efficiency [57]. This strategy involves engineering key proteins responsible for light capture, photoprotection, and energy transfer to enhance robustness under fluctuating light conditions. Lu et al. engineered a *hlr1* mutant in *Nannochloropsis oceanica*, leading to alterations in photosystem I (PSI) and a reduction in reactive oxygen species (ROS) production [30]. This improved high-light tolerance, demonstrating that target genetic modifications can enhance photosynthetic robustness. In addition, researchers found that certain enzymes in photosynthetic microorganisms, though not directly involved in photosynthesis, can impact pigment production. For example, the PhoD-type alkaline phosphatase (AP) gene influences both pigment synthesis and photosynthesis efficiency. Using CRISPR technology, Li et al. engineered the PhoD_45757 mutant (mPhoD44) in the diatom *Phaeodactylum tricornutum*. In comparison to the WT, this led to a 37% elevation in the content of Chl-a and a 49% increase in the content of chlorophyll c [31]. Another member of the AP family, PhoA, has shown similar effects. Zhang et al. created a PhoA mutant using CRISPR/Cas9, which reduced the enzyme’s organophosphate (DOP) hydrolase activity. However, this modification resulted in substantial augmentations in the levels of cellular pigments, carbon, and lipids. Moreover, it brought about improvements in the photosynthetic rate, growth rate, and the transcriptional levels of related metabolic pathways [32]. These studies illustrate how synthetic biology can reprogram photosynthesis at the protein level, allowing for enhanced light capture and photoprotection.

### 2.2. Transcriptional and Post-Transcriptional Regulation of Light-Harvesting Efficiency

Beyond direct protein modifications, transcriptional and post-transcriptional regulation plays a critical role in controlling LHC abundance and pigment composition in response to light conditions. Agarwal et al. identified the LHC-regulating Myb (LRM) transcription factor, which governs LHC gene expression in *Phaeodactylum tricornutum* [34]. Knocking down LRM impaired dynamic light absorption adjustments, highlighting the potential of transcriptional engineering for fine-tuning photosynthetic responses. NAB1, a nucleic acid-binding protein in *Chlamydomonas reinhardtii* (*C. reinhardtii*), regulates light-harvesting antenna complex expression [58]. Engineered strains with NAB1-controlled chlorophyllide a oxygenase (CAO) expression exhibited twice the biomass productivity, while maintaining optimal state transitions and photoprotection [33]. Regulating the size of the light-harvesting antenna enhances non-photochemical quenching (NPQ) processes and state transitions, increasing photosynthetic rates and significantly improving light energy utilization efficiency under low-light conditions, ultimately leading to greater biomass accumulation.

### 2.3. Metabolic and Hormonal Control of Photosynthetic Pigments

Synthetic biology approaches can enhance precursor availability for pigment synthesis, thereby improving light absorption and energy conversion. For instance, engineering carotenoid biosynthesis pathways has shown promise for improving photoprotection and minimizing oxidative damage, paving the way for metabolically optimized strains [59]. In addition to metabolic modifications, hormonal regulation also plays a critical role in modulating photosynthetic pigment levels. Zhang et al. demonstrated that the exogenous application of abscisic acid (ABA) downregulated the LHCA1 gene in *Nannochloropsis haitanensis*, reducing light capture and inhibiting photosynthesis [35]. This reduced light capture and photosynthetic performance, suggesting that phytohormone-responsive circuits could be engineered as synthetic regulators to optimize photosynthetic output. Together, these studies highlight that metabolic engineering and hormonal regulation provide additional mechanisms for optimizing photosynthetic efficiency by modulating pigment production and antenna size.

## 3. Adjustment of Electron Transport Systems

**Figure 3:**
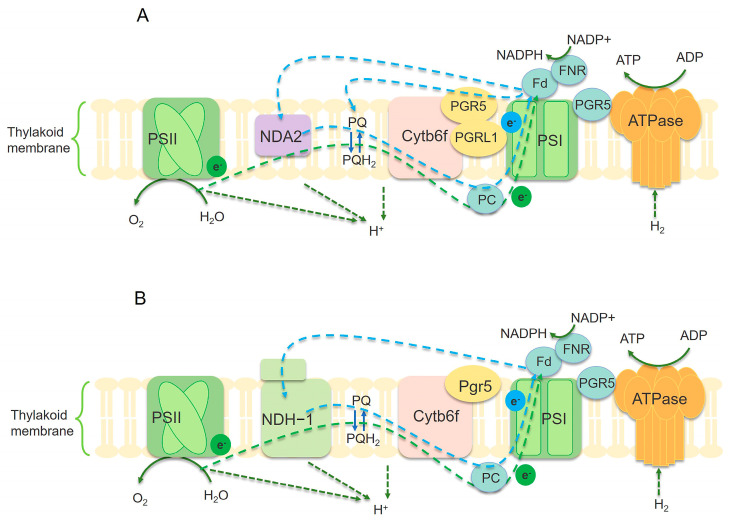
Illustration of electron transfer mechanism in microalgae (**A**) and cyanobacteria (**B**). The blue dashed arrow represents the circulating electron flow (CEF) around PSI. The green arc-shaped dashed line represents the linear electron flow (LEF) between PSII and PSI. NDH-1 complex mediates CEF in cyanobacteria (**B**). There is no NDH-1 in photosynthetic microalgae, replaced by NDA2 (**A**), and PGR5/PGRL1 also mediates CEF. PQ/PQH2: plastoquinone. PC: plastocyanin. Fd: ferredoxin. FNR: ferredoxin-NADP(H) reductase. Cytb6f: cytochrome b6-f complex [60,61].

The electron transfer process during photosynthesis represents a crucial stage. It encompasses two photosystems, namely Photosystem I (PSI) and Photosystem II (PSII). These two photosystems collaborate to transform light energy into chemical energy [60]. Adopting a systematic optimization approach for the electron transfer process may serve as an effective strategy to improve photosynthetic efficiency (Figure 3).

### 3.1. Regulation of Electron Transport Carriers

Plastoquinone (PQ) functions as a crucial electron carrier within both the photosynthetic and respiratory electron transport chains. In cyanobacteria and microalgae, it facilitates the conveyance of reducing power and energy [60]. In a study by Fan et al., when the enzyme geranylgeranyl diphosphate:4-hydroxybenzoate geranylgeranyl transferase (LepGT) was overexpressed in *Synechocystis* sp. PCC 6803 (*Synechocystis* 6803), the content of plastoquinone (PQ) decreased by 22.18%. Unexpectedly, such a reduction gave rise to an improvement in both the photosynthetic rate and the respiration rate [36]. Compared to the WT, the strain overexpressing LepGT showed an 11.55% increase in electron transport efficiency. NADP+ and NAD+ serve as indispensable electron acceptors within the realm of energy metabolism [36]. Specifically, NADP+ functions as an electron acceptor in the light-dependent reactions of photosynthesis. Moreover, its biosynthesis is of great significance for photoautotrophic growth [36]. By modulating PQ biosynthesis, the engineered *Synechocystis* strains exhibited enhanced electron transport efficiency, improved PSII activity, and optimized ATP/NADPH production, leading to an overall increase in photosynthetic rate and biomass accumulation.

### 3.2. Regulation of Photosystem Stoichiometry

PSI and PSII serve as indispensable constituents of the photosynthetic electron transport chain. The PSI core complex contains the reaction center pigment P700, which functions as an electron acceptor, and the PSI LHC, which transfers electrons from phycobilins to ferredoxin [60,62]. Alterations in the stoichiometry of Photosystem I (PSI) and Photosystem II (PSII), frequently occurring as a reaction to diverse light qualities, can act as a compensatory mechanism in the thylakoid membrane to equalize light absorption between the two photosystems [63]. Photosynthetic microorganisms can adjust the relative abundance of their light-harvesting complexes, leading to different PSI-to-PSII ratios, which can significantly impact the photosynthetic efficiency. Within the cyanobacterial strain *Synechocystis* 6803, PSI is typically 2 to 4 times more abundant than PSII [64]. In an experiment by Moore et al., researchers modified the promoter region of the psaAB operon to adjust the PSI-to-PSII ratios, creating strains with reduced PSI level. These modified strains demonstrated higher productivity under high-light intensities, as they reached saturation at these levels, optimizing their photosynthetic capacity [37]. The reduction in PSI content in *Synechocystis* 6803 led to enhanced light utilization, improved linear electron transport, and better growth under high-light conditions, ultimately increasing productivity. The BtpA protein (biogenesis of thylakoid proteins A) is known to regulate PSI levels post-transcriptionally by stabilizing the core PSI protein PsaA in *Synechocystis* 6803. In an associated study, Knoot et al. engineered a CRISPR interference (CRISPRi) system using dCas12a (dCpf1) to precisely control PSI levels in *Synechococcus* sp. UTEX 2973 (*Synechococcus* 2973). This system achieved over 90% inhibition of high-abundance gene targets in the absence of inducers. By targeting the homologous btpA gene in *Synechococcus* 2973, researchers reduced PSI levels to 13% of the control group1 mM. Additionally, the Chl-a content per cell in the modified strain dropped to less than 25% of that in the uninduced control. When IPTG was removed by refreshing the culture medium, PSI levels returned to normal within 48 h, indicating that PSI expression in these strains could be reversibly controlled [38]. Physical induction can also alter the PSII/PSI ratio. For instance, Balan et al. exposed *Chlorella vulgaris* to UVB radiation, which specifically stimulated PSII, increasing antenna size 10-fold. Under low-light laboratory conditions, a larger antenna size enables PSII to capture photons more efficiently, thereby promoting the enhancement of photosynthetic efficiency. This treatment produced a PSII-to-PSI ratio that was 2 to 7 times higher than that in control group strains grown solely under photosynthetically active radiation (PAR) at 5000 lux without UV exposure. Strains with an increased PSII/PSI ratio exhibited significantly higher photosynthetic efficiency and growth, achieving a maximum specific growth rate 4.3 times greater than that of the control group [39].

### 3.3. Regulation of Cyclic Electron Flow (CEF)

CEF, powered by PSI, synthesizes ATP without generating extra NADPH [65,66,67,68]. CEF operates primarily through two pathways, including the “antimycin A-insensitive” pathway [69], which involves the NADH dehydrogenase-like complex (NDH) (Figure 3B) [70,71], and the “antimycin A-sensitive” pathway, also known as the “PGR5-dependent CEF” pathway, involving the thylakoid proteins proton gradient regulation 5 (PGR5) and PGR5-like photosynthetic phenotype 1 (PGRL1) (Figure 3A) [72,73]. CEF plays a crucial role in preserving the redox equilibrium of the photosynthetic machinery and in precisely adjusting the NADPH/ATP ratio, particularly when light conditions are fluctuating [74]. According to research conducted by Jokel et al., the green alga *C. reinhardtii* was used to construct knockout mutants for the PGR5 and PGRL1 genes. The growth of the pgr5 mutant was severely impaired under mild fluctuating light (FL) conditions (20 μmol photons m^−2^ s^−1^ for 5 min, followed by 200 μmol photons m^−2^ s^−1^ for 30 s) and completely inhibited under more intense FL conditions (20 μmol photons m^−2^ s^−1^ for 5 min, followed by 600 μmol photons m^−2^ s^−1^ for 30 s). Under these conditions, both pgr5 and pgrl1 mutants showed varying degrees of growth inhibition. Immunoblot analysis indicated damage to the photosynthetic machinery, in the pgr5 mutant, the PsaA subunit of the PSI was reduced fivefold, while in the pgrl1 mutant, the Cyt f subunit of the Cyt b6f complex was reduced by 50%. Many cyanobacterial species have genes encoding PGR5-like proteins, but their role in photosynthesis remains incompletely elucidated [40]. As reported in a scientific investigation carried out by Margulis et al., overexpression of the pgr5 gene in *Synechocystis* 6803 led to a fivefold increase in chlorophyll accumulation and a corresponding increase in active PSI units, with the maximum P700 oxidation level (∆Amax) reaching approximately 250% that of the WT [41]. Conversely, Selão et al. knocked out pgr5 in *Synechococcus* sp. PCC 7002 (*Synechococcus* 7002), leading to an increased cellular [NADPH]/[NADP+] ratio and enhanced the NADP+-dependent lactate dehydrogenase (LDH) activity. This modification boosted the reduction in pyruvate to d-lactate, leading to nearly a sevenfold increase in d-lactate accumulation (≈1000 mg/L vs. ≈150 mg/L in the WT) under conditions of 25 °C and 0.04% (*v*/*v*) CO_2_ [42]. In *C. reinhardtii*, an additional NADPH-dependent and Fd-independent pathway involving the type II NAD(P)H dehydrogenase (NDA2) has been identified [75,76]. Baltz et al. successfully overexpressed the NDA2 gene in *C. reinhardtii*, resulting in a significant increase in hydrogen production via an indirect pathway, achieving almost twice the hydrogen production rate of the non-overexpressing strains. Although the PsaD subunit levels were reduced in the engineered strain, PSI activity remained unaffected. Additionally, in the dark, the reduction rate of P700^+^ was more rapid in the strain overexpressing NDA2 compared to that in the control, indicating that NDA2 overexpression effectively enhanced CEF activity [43].

## 4. Regulation of Carbon Assimilation

**Figure 4:**
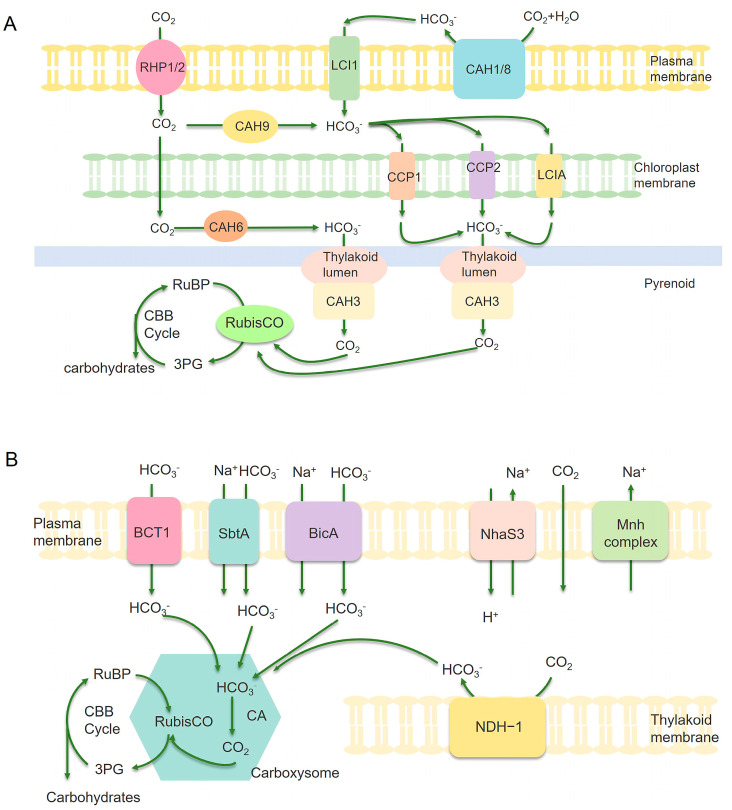
Illustration of carbon assimilation mechanism in microalgae (**A**) and cyanobacteria (**B**). (**A**) CAH1, CAH3, CAH6, CAH8, and CAH9 are various subtypes of carbonic anhydrase. Transport parking space RPH1/RPH2/LC1 on the plasma membrane and LCIA/CCP1/CCP2 located on the chloroplast envelope are Ci transporters. Arrows represent carbon assimilation pathways. (**B**) BCT1, SbtA, and BicA are bicarbonate transporters that can actively transport HCO_3_^−^ into the cytoplasm. The carboxysome is an important component of cyanobacteria CCM, which contains enzymes RuBisCO and CA inside. CA converts bicarbonate into CO_2_ in the carboxysome, thereby increasing the concentration of CO_2_ near the key enzyme RuBisCO and promoting CO_2_ conversion. NDH-1 complex can convert CO_2_ into HCO_3_^−^, which can prevent CO_2_ leakage from the carboxysome. RuBisCO catalyzes the reaction of Riboulose-1,5-bisphosphate (RuBP) with CO_2_ to produce 3-phosphoglycerate (3-PG), thereby achieving CO_2_ fixation [78,79].

Carbon assimilation is a key step in photosynthesis, through which photosynthetic microalgae convert inorganic carbon (CO_2_) into organic carbon (such as glucose and starch) (Figure 4). This process provides the energy and raw materials for their growth and development [77]. Enhancing carbon assimilation is crucial for improving biomass productivity, optimizing biofuel synthesis, and increasing photosynthetic efficiency. This section categorizes advancements in carbon assimilation into three major strategies, each focusing on a key regulatory mechanism.

### 4.1. Improving Ci (HCO_3_^−^/CO_2_) Uptake and Transport

Efficient carbon assimilation begins with the uptake and concentration of Ci (HCO_3_^−^/CO_2_). Cyanobacteria and microalgae utilize CO_2_-concentrating mechanisms (CCM) to actively capture CO_2_, thereby boosting photosynthetic rates. Engineering Ci uptake and transport systems has emerged as a promising strategy for improving CO_2_ fixation and biomass yield. For example, Gupta et al. examined the impact of two distinct ribosome binding site (RBS) sequences along with three different promoters (PcpcB, Pcpc560, and PrbcL2) on the expression of sodium-dependent bicarbonate transporter SbtA in *Synechococcus* 7002. The optimized construct using Pcpc560 and RBS2, resulted in a 90% increase in biomass under air or 1% CO_2_ conditions, demonstrating the effectiveness of transporter engineering for enhanced carbon uptake [44]. Wang et al. analyzed BicA, a bicarbonate transporter belonging to the SLC26 family, which plays a critical role in CCM in *Synechocystis* 6803. The deletion of ndhD3, ndhD4, cmpA, sbtA, and bicA resulted in growth arrest under both ambient CO_2_ conditions, highlighting their essential role in HCO_3_^−^ uptake and transport. However, the overexpression of BicA restored growth, demonstrating its potential for bioengineering CO_2_ uptake in heterologous hosts [45]. In *Synechococcus elongatus* PCC 7942 (*Synechococcus* 7942), a mutant strain engineered for α-farnesene production showed growth inhibition under ambient CO_2_ conditions due to the deletion of β-carboxysome and the CCM genes. The overexpression of carbonic anhydrase and bicarbonate transporters restored growth and increased α-farnesene production to 5.0 ± 0.6 mg/L, demonstrating that Ci uptake optimization contributed to the enhanced carbon flux for bioproduct synthesis [46]. These studies underscore the importance of Ci transporters in improving carbon capture, providing opportunities for synthetic biology applications in biofuel and biochemical production.

### 4.2. Optimizing Carbon Fixation Through RuBisCO and Carbonic Anhydrase Engineering

Ribulose-1,5-bisphosphate carboxylase/oxygenase (RuBisCO) assumes the responsibility of catalyzing the fixation of CO_2_ during the photosynthesis process. Notwithstanding its crucial function, RuBisCO is regarded as an enzyme with low efficiency, making it a primary target for endeavors in directed evolution [80,81,82]. Huang et al. demonstrated that the deletion of RuBisCO accumulation factor 1 (Raf1) in *Synechococcus* 7942 reduced RuBisCO RbcL8S8 holoenzyme by 30% and maximum CO_2_ fixation capacity by 39% [47]. Bolay et al. identified sll0998 (rbcR), a trans-acting transcriptional regulator, as a key modulator of RuBisCO gene expression in the *Synechocystis* 6803 [48]. The knockout of rbcR led to reduced RuBisCO expression, impaired growth, and decreased CO_2_ assimilation, indicating its regulatory role. Lu et al. knocked out SeXPK, a phosphoenolpyruvate carboxykinase in the *Synechococcus* 7942 strain, leading to a 63% increase in RuBisCO carbon fixation efficiency without affecting growth [49]. Carbonic anhydrase (CA) is an ancient zinc-containing metalloenzyme that catalyzes the interconversion between CO_2_ and HCO_3_^−^, playing a critical role in carbon assimilation and transport (Figure 4) [83]. Engineering CA activity provides an effective means to enhance CO_2_ conversion rates and optimize metabolic flux toward biomass accumulation. Fan et al. studied the effects of acetazolamide (a CA-specific inhibitor) on *Phaeodactylum tricornutum* and *Nannochloropsis oceanica*, finding significant reductions in growth rate and photosynthetic activity [50]. Lin et al. overexpressed MICA, a highly active CA isoform, in *Chlorella sorokiniana* and *Chlorella vulgaris*, leading to higher CO_2_ fixation rates, increased biomass yield, and enhanced lipid production [51]. Together, these studies demonstrated that engineering the activity of CA and RuBisCO provides an effective means to increase photosynthetic performance and enhance carbon flux toward biomass accumulation.

### 4.3. Exploring Photomixotrophic Cultivation for Greater Carbon Utilizaiton

Some microalgae and cyanobacteria can grow either photoautotrophically or photomixotrophically, simultaneously utilizing CO_2_ and organic carbon sources, like xylose, glucose, glycerol, or acetate [84]. The use of dual carbon sources enhances the growth and metabolic activity of photosynthetic microorganisms, offering attractive opportunities for biotechnology applications [85]. Although cyanobacteria can naturally utilize glucose, the central carbon metabolism leads to the release of carbon atoms in the form of CO_2_, resulting in a net loss of carbon. To address this, Song et al. engineered a synthetic nonoxidative cyclic glycolysis (NOG) pathway in *Synechocystis* 6803 to increase the carbon flux from glucose to the key intracellular precursor acetyl-CoA, successfully doubling the intracellular pool of the acetyl-CoA pool [52]. Moreover, to reduce competition for glucose, the native Entner–Doudoroff (ED) pathway was knocked out in the engineered strain carrying the NOG pathway, further increasing acetyl-CoA level by up to 280%. Xylose, which constitutes a significant part of lignocellulose and stands as the second-most prevalent sugar in nature following glucose, represents a promising renewable resource for the production of biofuels and chemicals [52]. Some model cyanobacteria have been engineered to utilize xylose by introducing a major facilitator superfamily transporter, XylE, alongside the native substrate, glucose, to enhance the production of heterologous ethylene and 2,3-butanediol [86,87]. More recently, Pressley et al. investigated the metabolic state of xylose-consuming mixotrophic *Synechococcus* 7942 during the production of 2,3-butanediol. They found that the overexpression of phosphoribulokinase (Prk) and ribulose-5-phosphate-3-epimerase (Rpe) led to a 29% increase in 2,3-butanediol titer, while the knockout of cp12 boosted production by 53% [53]. *C. reinhardtii*, recognized as a model photosynthetic organism, is also capable of mixotrophic growth, making use of supplementary organic carbon sources, which demonstrates its metabolic flexible incorporating diverse carbon intermediates [88]. Additionally, its potential in bioremediation and bioproduct synthesis has garnered increasing attention, as it can efficiently remove contaminants from wastewater, while also serving as a platform for biofuel and high-value compound production [89]. Yao et al. achieved formate tolerance exceeding 40 mM in *C. reinhardtii* through a combination of co-substrate addition, evolutionary approaches, and growth condition optimization. The study demonstrated that formate not only enhanced growth and photosynthetic activity but also increased biomass by 27% and photosynthetic oxygen evolution by 60% [54]. These findings suggest that photomixotrophic cultivation offers higher metabolic flexibility and greater carbon conversion efficiency, making it a promising strategy for industrial bioproduction.

## 5. Challenges of Regulating Photosynthesis Efficiency Through Synthetic Biology

Photosynthetic microorganisms, with their remarkable ability to absorb carbon dioxide and perform oxygen-producing photosynthesis, have emerged as a top choice for green biomanufacturing. By leveraging synthetic biology to regulate the photosynthesis process in these microorganisms, we can control their photosynthetic rate and growth and even modify them to produce high-value chemicals. For example, introducing exogenous synthetic pathways into cyanobacteria can enable the production of compounds, such as sucrose [9], inositol [10],3-HB,3-HV,3-HP [11], and astaxanthin [12]. Optimizing the photosynthetic processes of microalgae and cyanobacteria using synthetic biology holds significant potential for expanding their industrial applications in biomanufacturing. However, the complexity of photosynthesis, the unknown proteins involved in photosynthesis, and the limitations of engineering tools and ecological adaptation present significant challenges in leveraging synthetic biology to regulate photosynthesis.

### 5.1. Isolated Gene Engineering Insufficient to Achieve Measurable Improvements

In cyanobacteria or microalgae, modifying one or a few individual genes often has a limited impact on enhancing photosynthetic efficiency. This is due to the complex, multi-gene collaborative nature of photosynthesis, which involves interconnected processes, such as light capture, electron transfer, and carbon fixation. Altering a single component is unlikely to overcome constraints in other processes, thereby failing to achieve a significant improvement in overall photosynthetic efficiency. Melis demonstrated that, while reducing antenna size can enhance the photosynthetic efficiency of individual cells, this modification offers limited benefits for overall productivity under high-density culture conditions [22]. Kirst et al. deleted the TLA3-CpSRP43 gene in *C. reinhardtii*, leading to a reduction in antenna size. However, the anticipated increase in photosynthetic efficiency was not observed, suggesting that the impact of a single genetic modification may be limited [90]. In their article, Angermayr et al. emphasized that, while various genetic modifications have been applied to the metabolic pathways of cyanobacteria, the inherent complexity of photosynthesis means that altering a single or a few genes is typically insufficient to achieve significant improvements in photosynthetic efficiency. Instead, systematic and multi-level engineering strategies are required [7]. All these findings demonstrate that, in cyanobacteria or microalgae, modifying one or a few genes individually may result in only limited improvements in photosynthetic efficiency. A comprehensive approach that considers all aspects of photosynthesis and employs systematic optimization is essential.

### 5.2. Unknown Proteins Involved in Photosynthesis Emerged as Potential Factors Influencing the Regulation

Unknown proteins may play crucial roles in regulating photosynthesis in cyanobacteria and microalgae and serve as potential variables in synthetic biology design. For instance, an unannotated protein identified in *C. reinhardtii* appears to be implicated in the assembly and maintenance of the stability of the PSII supramolecular complex, with its mutation causing significant impairment of photosynthesis [91]. Recent studies have identified that small proteins in cyanobacteria play key roles in photosynthesis, contributing to cellular redox regulation, carbon/nitrogen balance, and various metabolic activities. For example, as Krauspe et al. discovered, in *Synechocystis* 6803, the diminutive protein NblD assumes a critical role in the degradation process of phycobilisomes during nitrogen-restricted circumstances. Simultaneously, it is indispensable for preserving the cellular quantities of amino acids, nitrogen-bearing metabolites, and organic acids [92]. The microalgal nitrogen-fixing bacterial consortia have been explored for bioremediation, biofuel production, and biofertilization applications. However, uncharacterized molecular components within these consortia play a role in regulating photosynthetic efficiency and nitrogen assimilation, which suggests that further research into unknown proteins may uncover new regulatory mechanisms [21]. Given the abundance of unknown proteins in microalgae and cyanobacteria, manipulating specific genes or pathways may reveal regulatory interactions between these unknown proteins and the target genes. Such interactions could significantly impact photosystem function, metabolic network balance, and biological adaptability.

### 5.3. Challenge of Balancing Cell Growth and Product Synthesis in Photosynthesis Regulation

Employing synthetic biology to regulate photosynthesis in microalgae and cyanobacteria holds potential to enhance photosynthetic efficiency and redirect energy and carbon flow toward target products. However, it may also impact cell growth and adaptation due to resource reallocation or metabolic burden [93]. Li et al. constructed ADP-glucose pyrophosphorylase-deficient mutants by inserting mutations, resulting in a lipid content 3.5 times higher than that of WT. However, at the same time, the growth of the mutants was damaged, and the PSII quantum yield was significantly lower than that of WT [94]. They also revealed that enhancing the lipid synthesis pathway in microalgae limits protein and pigment synthesis due to the preferential allocation of carbon and energy to lipid production. Anne M. et al. engineered the cyanobacterium *Synechococcus* 7942 through genetic knockout of the free fatty acid (FFA)-recycling acyl-ACP synthetase and heterologous expression of thioesterase to release FFAs. However, the engineered strain exhibited reduced photosynthetic yield, Chl-a degradation, and altered cellular localization of light-harvesting pigments, including phycocyanin and allophycocyanin [95]. Song et al. modified the central carbon metabolic route. They achieved this by incorporating a newly designed NOG-like module and eliminating a native ED pathway to boost the acetyl-CoA and lipid content, yet the cell growth in the engineered strain was remarkably decreased [52]. All these findings demonstrated the importance of collaboratively considering both cell growth and product synthesis when engineering photosynthetic cell factories.

### 5.4. Optimization Achieved in the Laboratory Proves Difficult to Reproduce in Large-Scale High-Density Cultivation

Translating genetic engineering advancements from laboratory-scale research to industrial-scale production presents significant challenges. These challenges primarily stem from strain instability, metabolic burden, process scalability, and bioreactor design constraints. Addressing these bottlenecks requires a multifaceted approach, integrating strain engineering, process optimization, and advanced bioreactor technologies. The key barriers to the commercial application of photosynthetic microalgae include regulatory constraints, strain instability in industrial settings, and economic feasibility. The genetic engineering strategy provides more insights for the large-scale production of photosynthetic microalgae, such as adaptive laboratory evolution, metabolic burden minimization, and process intensification strategies in bioreactor cultivation. Various types of photobioreactors have been developed for the cultivation of cyanobacteria and microalgae, each optimized for specific growth conditions and scalability. Bubble column photobioreactors feature a cylindrical structure divided into aerated and non-aerated zones to mimic airlift operation (7–24 cm in diameter). These reactors are typically illuminated by fluorescent tubes or LEDs distributed peripherally, requiring transparent materials (e.g., glass) for external lighting, while internal light sources allow the use of opaque materials. Stirred tank reactors rely on external or internal light sources (e.g., LEDs or optical fibers), with diameter optimization ensuring sufficient light intensity at the center. Optical fibers can couple natural and artificial light to adapt to diurnal variations, while red LEDs, due to their low energy consumption, are employed in wastewater treatment. Tubular reactors consist of a solar receiver (long tubes or helical structures optimized for light capture) and an airlift system (degassing the bubble column for oxygen removal), often illuminated by fluorescent lamps in laboratory settings. Airlift reactors utilize gas-driven circulation (via riser and downcomer zones) combined with external natural light, embedded LEDs, or hybrid lighting to balance efficient mass transfer and uniform light distribution. Each reactor type is tailored through differentiated designs (e.g., light source arrangement, structural optimization) to meet microalgal cultivation needs, spanning from laboratory pre-culture to industrial-scale production [96,97]. Nonetheless, when a single-species cultivation method is adopted to yield a high-density cell mass or the peak titers of particular products in photobioreactors, the over-absorption of photons by the cells in the top layer may prevent the light from reaching the cells deeper down [22]. For instance, Li et al. engineered a β-caryophyllene synthesis pathway in *Synechococcus* 2973, optimizing the expression of key synthases and precursor supply. In shake flask experiments, β-caryophyllene production reached approximately 121.22 μg/L. However, during high-density cultivation in a photobioreactor, although the yield increased to about 212.37 μg/L, the improvement was marginal compared to low-density cultures, suggesting that the benefits of enhanced light capture are constrained under high-density conditions [98].

## 6. Perspectives and Future Direction

### 6.1. AI-Guided Metabolic Engineering for Photosynthetic Optimization

**Figure 5:**
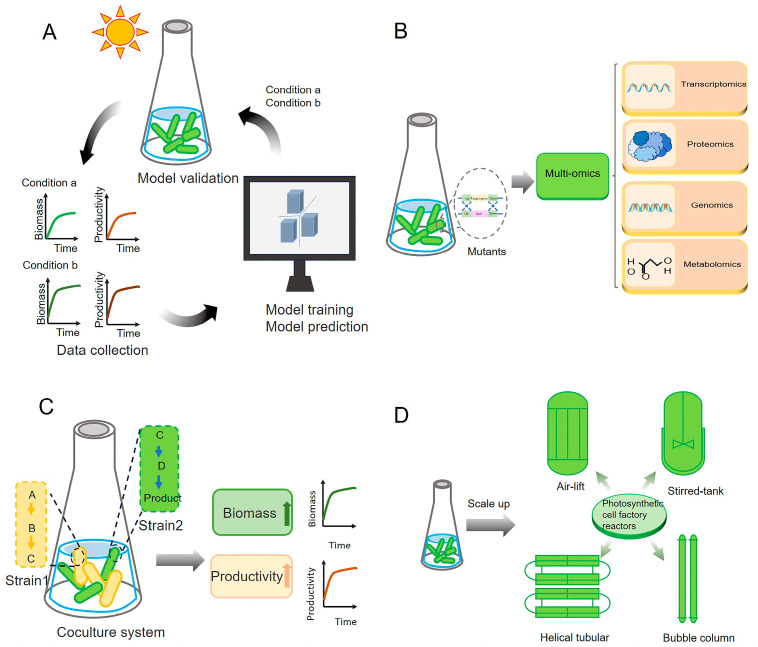
Prospects and further directions for regulating photosynthesis through synthetic biology. (**A**) Optimizing photosynthesis with artificial intelligence. Critical data, such as growth conditions, photosynthetic efficiency, and production yields, are collected using advanced sensors and monitoring systems. AI algorithms analyze the collected data to construct comprehensive system models, which predict optimal conditions and intervention strategies for enhancing photosynthesis, guiding experimental designs and engineering efforts. (**B**) Characterization of unknown proteins. Mutants deficient in specific proteins can be constructed and analyzed using integrative omics approaches to identify and characterize previously unknown proteins that may be involved in photosynthesis. (**C**) Construction of artificial coculture systems. Artificial coculture systems can be designed to distribute the metabolic burden of microalgal cell factories across different strains. This strategy simultaneously enhances both photosynthetic efficiency and product yield. (**D**) Development of photosynthetic cell factory reactors. By integrating data from cell factory cultivations at various scales, tailored designs for large-scale photosynthetic cell factory bioreactors can be developed, further enhancing the advantages of high-density cultivation.

With the aid of artificial intelligence, particularly machine learning, synthetic biology can identify regulatory targets within complex biological systems, enabling the precise optimization of microalgal biotransformation processes and cyanobacterial metabolic regulation (Figure 5A). Through the analysis of available data, machine learning algorithms are capable of calculating the most suitable environmental and light conditions for the cultivation of microalgae. They can also forecast biofuel yields, enhance energy conversion efficiency, and guarantee a higher-quality biofuel [99]. Several studies have demonstrated the feasibility of AI-based tools for improving photosynthetic efficiency. Kugler et al. combined constraint-based metabolic modeling and machine learning to identify key bottlenecks limiting CO_2_ fixation in *Synechocystis* 6803. Their model accurately predicted rate-limiting steps in the CBB cycle, enabling the rational engineering of more efficient CO_2_ assimilation pathways [100]. Long et al. applied AI algorithms to optimize light intensity and nutrient availability in microalgal cultures. The system successfully increased biomass productivity by 32% compared to conventional manual optimization [101]. By applying decision tree algorithms, Ahmet Coşguna et al. determined 11 sets of conditions that foster high microalgal productivity and 13 factors that could boost lipid content [102]. Machine learning tools can additionally function as a precious conduit for comprehending cellular physiology and forecasting rate-limiting steps within genome-scale metabolic networks. Consequently, they offer guidance for the bioengineering of cyanobacteria. All the discoveries derived from machine learning offer a pathway to gain insights into cellular physiology and anticipate the rate-limiting steps in metabolic networks. This, in turn, provides guidance for those engaged in the biomanufacturing of cyanobacteria.

### 6.2. Unveiling the Functions of Unknown Proteins via Integrated Approach

Understanding the functions of unknown proteins provides deeper insights into the photosynthetic mechanisms of cyanobacteria and microalgae. This knowledge facilitates the optimization of critical processes, such as light reactions, carbon fixation, and photoprotection. In addition to enhancing photosynthetic efficiency, it paves the way for innovative approaches to bioenergy and bioproduct synthesis. System modeling enables the prediction of unknown protein functions by integrating data from genomics, proteomics, transcriptomics, and metabolomics (Figure 5B). Recent omics-based studies have started to uncover novel proteins that influence photosynthetic performance. Nishiguchi et al. used multi-omics analysis to identify previously unknown proteins involved in carbon assimilation in *Synechocystis* 6803. Their findings provided a functional basis for metabolic rewiring strategies to enhance CO_2_ fixation [103]. Based on their model predictions, they successfully engineered a high ethanol-yielding strain of *Synechocystis* 6803, achieving an ethanol production of approximately 120 mg/L [103]. Using omics technologies, functional prediction, and experimental validation to uncover the roles of unknown proteins can greatly enhance the predictability and success rates of synthetic biology projects. However, the lack of comprehensive large datasets and accurate ORF prediction tools for small prokaryotic proteins poses significant challenges to identifying unknown small proteins and regulatory factors. To overcome these obstacles, integrating proteogenomic analysis with ribosome profiling offers a powerful approach to discovering more small proteins [104]. Additionally, applying machine learning to identify unknown proteins and regulatory factors holds great potential for providing valuable insights for synthetic biology [105].

### 6.3. Enhancing Microbial Synergy via Building Artificial Coculture Systems

Microbial consortia—engineered communities of photosynthetic microbes and heterotrophic bacteria—offer enhanced metabolic exchange, nutrient recycling, and CO_2_ fixation compared to monocultures (Figure 5C). Engineering microbial consortia can overcome metabolic bottlenecks in single-strain systems by distributing the metabolic load across multiple strains, reducing the burden on any one strain for both growth and product synthesis [106]. Artificial coculture systems can be classified into several types based on functional interactions, including photoautotroph–heterotroph consortia, diazotrophic cyanobacteria–microalgae consortia, and microalgae–yeast/bacteria consortia. Several successful synthetic coculture models have been experimentally validated for biotechnological applications. Zhang et al. constructed an artificial cyanobacteria–*E. coli* coculture system, where the engineered *Synechococcus* 2973 provided fixed carbon for *E. coli*, enabling the production of 3-hydroxypropionic acid (3-HP) [9]. Xu et al. constructed a microalgae–yeast coculture system and found that both biomass and lipid yield were increased [107]. In addition, the artificial coculture system demonstrates excellent robustness and greater adaptability to changing environments. Take an example, Heys et al. found that various coculture systems, formed by combining sucrose-secreting cyanobacteria with three different heterotrophic bacteria (*Saccharomyces cerevisiae*, *E. coli*, or *Bacillus subtilis*), can be successfully established, demonstrating the robustness of autotrophic–heterotrophic mixed bacterial systems [108]. Moreover, Kruyer et al. demonstrated an artificial photosynthetic coculture system that produces rocket fuel and is capable of adapting to the harsh conditions of the Martian environment [109], a finding that was later confirmed by Ramalho et al. [110]. Diazotrophic cyanobacteria, such as *Anabaena* spp. and *Nostoc* spp., can fix atmospheric nitrogen, providing a sustainable nitrogen source for microalgae growth without the cost of chemical fertilizers [21]. The artificial coculture systems are likely to be further optimized through the careful consideration of metabolic compatibility, nutritional requirements, and environmental conditions, paving the way for broader applications.

### 6.4. Designing Photosynthetic Cell Factory Reactors for Large-Scale Production

The design of photosynthetic cell factory reactors is a critical step in optimizing photosynthesis, enhancing target product yields, and enabling sustainable production (Figure 5D). In practical applications, maintaining the stability and efficiency of photosynthetic cell factories in large-scale cultivation remains a critical challenge that needs to be addressed. For the study of photosynthesis in microalgae and cyanobacteria, the photobioreactor (PBR) represents a key technological advancement [111]. With the advancement of sensors, Internet of Things (IoT) technologies, and artificial intelligence (AI), future photobioreactors will become increasingly intelligent. They will possess the ability to conduct the real-time surveillance and modification of environmental variables, including temperature, light intensity, pH value, CO_2_ concentration, oxygen content, and more, ensuring that microalgae or cyanobacteria can perform photosynthesis under optimal growth conditions [112]. An automated control system can be established based on the growth cycle, dynamically optimizing parameters, such as gas input, light intensity, and temperature, based on real-time data to maximize biomass production and photosynthetic efficiency. In addition, future photobioreactors should focus more on the recovery and utilization of CO_2_, particularly in the treatment of industrial waste gases. The photosynthesis of microalgae and cyanobacteria can be harnessed to absorb and convert CO_2_, helping to reduce greenhouse gas emissions. Gaseous industrial wastes, for instance, those emitted by power plants, steel mills, and fertilizer plants, can be introduced into the photobioreactor, where microalgae’s photosynthesis transforms CO_2_ into biomass [113]. By optimizing the concentration and dissolution of CO_2,_ the photosynthetic efficiency of microalgae can be further enhanced, particularly through the precise control of gas concentrations in closed reactors.

## 7. Conclusions

Photosynthetic microorganisms, such as microalgae and cyanobacteria, are attracting growing interest due to their prospects in eco-friendly biomanufacturing. Recent advancements in synthetic biology have successfully enhanced photosynthetic efficiency by engineering light-harvesting complexes, optimizing electron transport, and improving carbon fixation pathways. Experimental studies have demonstrated that targeted modifications, such as the rational design of antenna complexes, transcriptional control of photosynthetic genes, and metabolic engineering of carbon flux, can lead to measurable improvements in biomass productivity and biofuel yield. For instance, engineered strains of *C*. *reinhardtii* with optimized light-harvesting antenna sizes have exhibited double the biomass productivity of wild-type strains [33]. In cyanobacteria, the application of constraint-based modeling combined with machine learning has identified rate-limiting steps in the CBB cycle, leading to improved carbon fixation efficiencies [100]. Moreover, synthetic microbial coculture systems have demonstrated enhanced growth rates and product titers, as seen in engineered cyanobacteria-*E. coli* consortia that increased 3-HP production under photoautotrophic conditions [9]. While these successes illustrate the potential of synthetic biology, challenges remain, including the functional characterization of unknown proteins involved in photosynthesis and the optimization of growth-product trade-offs. The integration of AI-driven metabolic modeling and real-time bioprocess control is beginning to refine experimental design and strain engineering, though further validation is needed to confirm long-term scalability and industrial feasibility. As synthetic biology continues to merge with AI, multi-omics approaches, and advanced bioreactor designs, future developments will likely focus on the precise regulation of photosynthetic pathways, enhanced stress tolerance, and more efficient bioproduction systems. These innovations will not only boost the economic viability of photosynthetic biomanufacturing but also contribute to sustainable carbon capture and bioresource utilization.

## Figures and Tables

**Table 1:** Regulation of photosynthesis in microalgae and cyanobacteria by synthetic biology.

Regulatory Targets	Strains	Strategies and Tools for Regulating Photosynthesis	Influence on Photosynthesis	Impact	Ref.
Protein engineering for light absorption optimizaiton	*Nannochloropsis oceanica*	Knocking out the high light resistance 1 (HLR1) gene using CRISPR-Cas9 and RNAi methods	A reduction in the PSI antenna size and a decrease in ROS production, resulting in improved tolerance to high-light conditions	Enhance high-light tolerance	[30]
	*Phaeodactylum tricornutum*	Knocking out the PhoD-type alkaline phosphatase (AP) gene using CRISPR	In the mutant mPhoD44 cells under P-enrichment, the Chl-a and Chl-c contents increased by 37% and 49%, respectively, compared to the WT	Elevation of photosynthetic pigment levels	[31]
	*Phaeodactylum tricornutum CCAP 1055/1*	Generating a functional knockout mutant of AP (PhoA) using CRISPR/Cas9	An increase in pigment, carbon, and lipid contents, alongside enhanced photosynthetic and growth rates, as well as elevated transcription levels of the associated metabolic pathways	Elevation of photosynthetic pigment levels	[32]
Transcriptional and post-transcriptional regulation of light-harvesting efficiency	*Chlamydomona reinhardtii*	Expressing a CAO gene extended by 50 mRNA bases that encode a binding site to inhibite the Nab1 translationa in *Chlamydomonas reinhardtii* CAO (chlorophyllide a oxygenase) gene knockout cell line	The photosynthetic rate significantly increased, with biomass productivity doubling that of the WT	Enhancement of photosynthetic rate	[33]
	*Phaeodactylum tricornutum*	Disrupting the function of the LRM transcription factor using antisense RNA interference	Unable to respond to changes in light intensity	Impairment of photoprotective capacity	[34]
Metabolic and hormonal control of photosynthetic pigments	*Nannochloropsis haitanensis*	Exogenous application of abscisic acid followed by analysis using transcriptomic techniques	Downregulation of the light-harvesting protein gene (LHCA1) leads to a reduced light-harvesting capacity	Reduction in light absorption	[35]
Regulation of electron transport carriers	*Synechocystis* sp. PCC 6803	Overexpressing 4-hydroxybenzoate geranyltransferase (lepgt)	The electron transfer efficiency increased by up to 111% times compared to the WT	Enhancement of photosynthetic electron transport efficiency	[36]
Photosystem stoichiometry	*Synechocystis* sp. PCC 6803	Introducing random nucleotides into psbA2 promoter by mutagenesis	Reducing PSI to enhance its yield under high-light conditions	Enhance high-light tolerance	[37]
	*Synechococcus* sp. UTEX 2973	Inhibiting *BtpA* gene via dCas12a (dCpf1) CRISPRi	Reducing PSI levels and intracellular Chl-a content	Reduction in light absorption	[38]
	*Chlorella vulgaris*	Ultraviolet (UV) mutagenesis	Increasing the PSII/PSI ratio by 2 to 7 times	Enhancement of photosynthetic efficiency under low-light conditions	[39]
Cyclic electron flow	*Chlamydomonas reinhardtii*	Generating pgr5 mutant by DNA insertional mutagenesis	Modifying the adaptability of mutants to fluctuating light conditions	Impairment of adaptation to fluctuating light conditions	[40]
	*Synechocystis* sp. PCC 6803	Overexpressing *pgr5* gene using pTKP2031v vector	Increasing chlorophyll accumulation, active PSI units, and the maximum P700 oxidation level	Elevation of photosynthetic pigment levels	[41]
	*Synechococcus* sp. PCC 7002	Knocked out the *pgr5* gene	Enhancing chlorophyll accumulation, active PSI units, the maximum P700 oxidation level (ΔAmax), the intracellular [NADPH]/[NADP+] ratio, and increasing d-lactate accumulation in the mutant strain by up to 700%	Elevation of photosynthetic pigment levels	[42]
	*Chlamydomonas reinhardtii*	Overexpressing NDA2 gene	Increasing the CEF activity and hydrogen production	Enhancement of CEF activity	[43]
Improving Ci (HCO_3_^−^/CO_2_) uptake systems and transport	*Synechococcus* sp. PCC 7002	Overexpressing Sbta through optimizing RBS sequences and promoters	Increasing the biomass by 90% compared to the WT	Enhancement of biomass accumulation	[44]
	*Synechocystis* sp. PCC 6803	Engineering the transporters for CO_2_ and HCO_3_^−^	Modulating the assimilation of HCO_3_^−^ to enhance cell growth	Enhancement of biomass accumulation	[45]
	*Synechococcus elongatus* PCC 7942	Overexpressing CA and BicA in an α-farnesene-producing strain lacking CCM	Enhancing α-farnesene production and improving adaptability to high CO_2_ conditions	Enhancement of elevated CO_2_ tolerance	[46]
Optimizing carbon fixation through RuBisCO and carbonic anhydrase engineering	*Synechococcus elongatus* PCC 7942	Knocking out Rubisco accumulation factor 1, *Raf1*	Reducing CO_2_ fixation rate and biomass accumulation	Reduction in biomass accumulation	[47]
	*Synechocystis* sp. PCC 6803	Partial knocking out *rbcR* gene	Downregulating gene expression of RuBisCO and its chaperone proteins rbcLXS, as well as the ccmK2K1LMN operon, which encodes carboxysome components	Reduction in carbon fixation capacity	[48]
	*Synechococcus elongatus* PCC 7942	Knocking out phosphoketolase (Sexpk)	Enhancing the CO_2_ fixation rate	Enhancement of carbon fixation capacity	[49]
	*Phaeodactylum tricornutum*, *Nannochloropsis oceanica*	Exogenous addition of the exCA-specific inhibitor acetazolamide	Inhibits the net photosynthetic rate, chlorophyll a content, and growth rate	Reduction in growth rate	[50]
	*Chlorella sorokiniana*, *Chlorella vulgaris*	Introducing MICA from *Mesorhizobium loti*	Increasing the lipid content by up to 220% compared to WT	Enhancement of lipid content	[51]
Exploring photomixotrophic cultivation for greater carbon utilizaiton	*Synechocystis* sp. PCC 6803	Introducing synthetic NOG pathway and knocking the native ED pathways	Increasing the intracellular pool of acetyl-CoA by approximately 280%	Enhancement of acetyl-CoA content	[52]
	*Synechococcus elongatus* PCC 7942	Overexpressing ribulose-5-phosphate-3-epimerase (Rpe) and phosphoribulokinase (Prk), as well as knocking out cp12	Increasing 2,3-butanediol production by 53%	Enhancement of biomass accumulation and 2,3-butanediol production	[53]
	*Chlamydomonas reinhardtii*	Laboratory evolution	Increasing tolerance to formate, biomass production, and photosynthetic oxygen evolution	Enhancement of biomass accumulation	[54]

## Data Availability

All the data have been submitted, and there is no other available data and materials.

## Abbreviations

The following abbreviations are used in this manuscript:

3-HB	3-hydroxybutyric acid
3-HV	3-hydroxyvaleric acid
3-HP	3-hydroxypropionic acid
TAG	Triglycerides
ATP	Adenosine triphosphate
LHCs	Light-harvesting pigment–protein complexes
LHCRs	Chlorophyll a-binding proteins
PSI	Photosystem I
ABA	Abscisic acid
NAB1	Nucleic acid binding 1
CAO	Chlorophyllide *a* oxygenase
AP	Alkaline phosphatase
PSII	Photosystem II
PQ/PQH_2_	Plastoquinone
DOP	Organophosphate
CCCP	Carbonyl cyanide m-chlorophenyl hydrazone
TCA cycle	Tricarboxylic acid cycle
CRISPRi	CRISPR interference
dCpf1	dCas12a
PAR	Photosynthetically active radiation
CEF	Cyclic electron flow
NDH	NADH dehydrogenase-like complex
PGR5	Proton gradient regulation 5
PGRL1	PGR5-like photosynthetic phenotype 1
FL	Fluctuating light
NDA2	Type II NAD(P)H dehydrogenase
Ci	Inorganic carbon
RBS	Ribosome binding site
CCM	CO_2_-concentrating mechanisms
CA	Carbonic anhydrase
CCMs	Carbon-concentrating mechanisms
Chl-a	Chlorophyll-a
RuBisCO	Ribulose-1,5-bisphosphate carboxylase/oxygenase
Raf1	RuBisCO accumulation factor 1
BN-PAGE	Blue native polyacrylamide gel electrophoresis
NOG	Nonoxidative cyclic glycolysis
ED	Entner-doudoroff
Rpe	Ribulose-5-phosphate-3-epimerase
Prk	Phosphoribulokinase
PBR	Photobioreactor
IoT	Internet of Things
LEF	Linear electron flow
PC	Plastocyanin
Fd	Ferredoxin
FNR	Ferredoxin-NADP(H) reductase
Cytb6f	Cytochrome b6-f complex.
